# Analysis of the Origin and Dissemination of HIV-1 Subtype C in Bulgaria

**DOI:** 10.3390/v14020263

**Published:** 2022-01-27

**Authors:** Ivailo Alexiev, Carla Mavian, Taylor Paisie, Massimo Ciccozzi, Reneta Dimitrova, Anna Gancheva, Asya Kostadinova, Carole Seguin-Devaux, Marco Salemi

**Affiliations:** 1 National Reference Laboratory of HIV, National Center of Infectious and Parasitic Diseases, 1233 Sofia, Bulgaria; naydenova.reneta@gmail.com (R.D.); gancheva.anna@gmail.com (A.G.); deshova.asi@gmail.com (A.K.); 2 Department of Pathology, Immunology, and Laboratory Medicine, University of Florida College of Medicine, Gainesville, FL 32610, USA; cmavian@ufl.edu (C.M.); tpaisie@ufl.edu (T.P.); salemi@pathology.ufl.edu (M.S.); 3 Emerging Pathogens Institute, University of Florida, Gainesville, FL 32610, USA; 4 Unit of Medical Statistics and Molecular Epidemiology, University Campus Bio-Medico, 00128 Rome, Italy; m.ciccozzi@unicampus.it; 5 Department of Infection and Immunity, Luxembourg Institute of Health, L-4354 Luxembourg, Luxembourg; carole.devaux@lih.lu

**Keywords:** HIV, subtype C, molecular epidemiology, Bulgaria

## Abstract

HIV-1 subtype C is the most abundant strain of HIV-1 infections worldwide and was found in the first known patients diagnosed with HIV/AIDS in Bulgaria in 1986. However, there is limited information on the molecular-epidemiological characteristics of this strain in the epidemic of the country. In this study, we analyze the evolutionary history of the introduction and dissemination of HIV-1 subtype C in Bulgaria using global phylogenetic analysis, Bayesian coalescent-based approach, and molecular clock methods. All available samples with HIV-1 subtype C from individuals diagnosed with HIV/AIDS between 1986 and 2017 were analyzed. Men and women were equally represented, and 24.3% of patients reported being infected abroad. The global phylogenetic analysis indicated multiple introductions of HIV-1 subtype C from various countries of the world. The reconstruction of a Bayesian time-scaled phylogenies showed that several Bulgarian strains segregated together in clusters, while others were intermixed in larger clades containing strains isolated from both European and non-European countries. The time-scale of HIV-1 subtype C introductions in Bulgaria demonstrates the early introduction of these viruses in the country. Our in-depth phylogenetic and phylogeographic analyses are compatible with a scenario of multiple early introductions in the country followed by limited local distribution in the subsequent years. HIV-1 subtype C was introduced in the early years of the epidemic, originating from different countries of the world. Due to the comprehensive measures for prevention and control in the early years of the epidemic in Bulgaria, HIV-1 subtype C was not widely disseminated among the general population of the country.

## 1. Introduction

Human immunodeficiency virus type 1 (HIV-1) is divided into four major phylogenetic groups: M (major), N (new), O (outlier), and P, representing independent cross-species transition from SIV in chimpanzee (Pan troglodytes (Ptt)) to HIV in humans [1,2]. HIV-1 group M, responsible for the current pandemic, comprises numerous genetically distinct subtypes (A–D, F–H, J, and K) as well as circulating recombinant forms (CRFs) and unique recombinant forms (URFs) [3,4,5,6,7,8,9,10]. The unequal worldwide distribution of different HIV-1 subtypes and CRFs has been explained by different founder effects followed by regional spread driven by socioeconomic and behavioral (circulation within specific risk groups) factors [4,5,6,7,8,9,10,11], in some cases sustained by the continuous inflow of new infections from neighboring geographic areas [12]. Subtype B is predominant in the Americas, Western Europe, and Australia, and subtype A prevails in Eastern Europe and Central Asia, including Russia and the former Soviet Union countries [3,6,12,13,14]. Subtype C accounts for about 46.6% of HIV-1 infections worldwide and is the most abundant strain in South Africa and Southeast Asia, followed by subtypes A and B [13,15]. CRFs and URFs are widely distributed in Central Africa and in countries where different subtypes co-circulate [13,16,17,18].

HIV-1 epidemic in the Balkan region, including Bulgaria, has been affected by various historic and socio-economic factors. The first HIV/AIDS case in Bulgaria was found in an individual infected abroad [19,20,21]. Our previous reports indicated that various HIV-1 subtypes and recombinant forms were introduced from various countries of the world and subsequently disseminated unequally among individuals from different transmission groups in Bulgaria, including heterosexuals (HET), men who have sex with men (MSM), and persons who inject drugs (PWID) [21,22,23]. Due to random founder events, multiple HIV-1 strains have had a chance to spread rapidly after being introduced into vulnerable groups. Indeed, such events have been observed since 2005, when two different CRFs (CRF01_AE and CRF02_AG) were independently introduced and rapidly disseminated among two geographically distinct subgroups of PWID, resulting in local outbreaks [19,24]. In contrast, other clades have been characterized by limited dissemination patterns and, after being passed on to few or single partners outside the risk populations, have reached a dead-end [21]. Such multiple introductions of different viral strains from abroad have led to a wide variety of circulating HIV-1 subtypes unevenly distributed among the populations of the country, and more than half of them are not the predominant-to-Europe subtype B [24,25].

HIV-1 subtype C was isolated from the first known patients diagnosed with HIV/AIDS in Bulgaria during the early years of the epidemic circulating in the HET population and thus differed from those in Western Europe [21]. The further spread of these strains was fueled by the continuous introduction of new viral variants from different parts of the world and subsequently distributed unevenly in geographical regions of the country. Until today, unlike subtype B, CRF01_AE, and CRF02_AG, which are mainly circulating among MSM and PWIDs, subtype C has only limited prevalence predominantly within HET populations [24]. Here, we present the first in-depth evolutionary history of the introduction and dissemination of HIV-1 subtype C in Bulgaria using the Bayesian coalescent-based approach and molecular clock methods.

## 2. Materials and Methods

### 2.1. Study Design and Specimen Preparation

In this nationwide study, all available blood samples from 37 individuals diagnosed with HIV-1 subtype C between 1986 and 2017 were collected during clinical follow-up at the National Reference Confirmatory Laboratory of HIV (NRCL of HIV) Sofia, Bulgaria. Demographic and epidemiological information were collected using patient self-assessment interviews following national regulations. Plasma samples were stored at −80 °C and linked to demographic and clinical data through an anonymous numerical code following the ethical standards of Bulgaria and information on the geographic origin, and sampling date were retained as previously described [21,22].

### 2.2. Sequence Analysis and Data Set

The HIV-1 *pol* gene was sequenced using the Viroseq HIV-1 Genotyping Test (Abbott, Chicago, IL, USA) and/or TruGene DNA Sequencing System (Siemens Healthcare, Erlangen, Germany) and either the Applied Biosystems 3130xl genetic analyzer or an OpenGene DNA sequencing system following the manufacturer’s protocol [24]. HIV-1 subtype C of the analyzed sequences was determined using the automated tool COMETv2.2 [26], REGA HIV-1 subtyping tool version 3.0 [27], and jpHMM [28] as well as by manual phylogenetic analysis using reference sequences downloaded from the Los Alamos sequence database (http://www.hiv.lanl.gov/, accessed on 22 December 2021). Phylogenetic relationships of the 37 Bulgarian sequences with 39 HIV-1 subtype reference sequences were inferred using the maximum likelihood (ML) method in IQ-TREE v1.6.11 Web Server. Stability of the ML tree topologies was tested using ultrafast bootstrap (1000 replicates) [29,30]. For the implementation of the extended phylogenetic analysis, all available HIV-1 subtype C sequences with known sampling year and country of origin were downloaded from the Los Alamos sequence database (http://www.hiv.lanl.gov/). Individual BLAST searches of all 37 Bulgarian sequences were performed, and the most similar GenBank sequences were retrieved for further analysis. The sequences were downloaded and then selected on the basis of the following inclusion criteria: (1) sequences already published in peer-reviewed journals (except for the new sequences described below); (2) no uncertainty about subtype assignment; (3) exclusion of putative recombinant sequences; and (4) known sampling time and city/state of origin were clearly established in the original publication.

Sequence alignments containing the Bulgarian sequences, BLAST search sequences, and the worldwide representatives from the Los Alamos database were performed using the MUSCLE algorithm implemented in AliView version 1.23 [31,32]. Additional quality control of the subtype purity and the possible presence of sequence gaps was performed. Duplicate sequences were removed for computational efficiency. After the clean-up and preliminary quality analysis, the complete dataset (Dataset-1) contained 1768 sequences, including the 37 new Bulgarian isolates. Twenty-three codons with drug-resistance mutation (detected by Stanford University HIV drug-resistance database available from https://hivdb.stanford.edu/, accessed on 22 December 2021) were manually removed from the alignment to avoid the confounding factor of convergent evolution in the phylogeny inference. The final alignment, comprised of 723 nucleotides in length (HXB2 positions 2280 → 3242), is available from the authors upon request. All Bulgarian HIV-1 subtype C sequences were deposited in GenBank with the following accession numbers: EF517409, JQ259060, EF517413, KJ765393, EF517416, KJ765400, KJ765476, KJ765556, KJ765557, JQ259067, EF517445, JQ259079, EF517468, KJ765568, KJ765574, JQ259151, KJ765434, and OK587312-OK587331.

For the subsequent analysis, two datasets referred to as Dataset-1 and Dataset-2 were used. Dataset-1, including all 1768 sequences, was used to infer an initial ML phylogenetic tree, with IQ-TREE (available from http://www.iqtree.org/, accessed on 22 December 2021) and the best-fitting model of nucleotide substitution [29,30], to investigate the relationship between Bulgarian sequences and HIV-1 subtype C strains isolated worldwide. Since the new Bulgarian strains did not cluster within a single monophyletic clade (see Results), implying multiple transmission events from foreign countries at different time points, Dataset-2, a reduced dataset of 300 sequences, was also generated to investigate HIV-1 subtype C phylodynamic patterns in-depth with the Bayesian coalescent framework implemented in BEAST v1.8.3 (http://beast.bio.ed.ac.uk/, accessed on 22 December 2021) [33,34]. After conducting advanced pre-analysis for the Dataset-2, the subset included 263 randomly selected HIV-1 subtype C sequences phylogenetically close to the Bulgarian strains in the initial ML tree as well as Bulgarian strains.

### 2.3. Likelihood Mapping and Temporal Signal Analysis

The phylogenetic and temporal signals were evaluated for Dataset-2 intended for evolutionary rate estimates and time-scaled phylogeny reconstruction. The phylogenetic signal was investigated by likelihood mapping analysis of 10,000 random quartets generated using the TreePuzzle 5.3 program [35]. The presence of a temporal signal in Dataset-2, necessary for the calibration of a reliable molecular clock (see next section), was assessed by inferring an ML phylogenetic tree with IQ-TREE program and analyzing the linear correlation between tip-to-root distances and sampling time of each strain in the tree with the TempEst v1.5.1 software (available from http://tree.bio.ed.ac.uk/software/tempest/, accessed on 22 December 2021) [36].

### 2.4. Bayesian Phylogenetics and Molecular Clock Calibration

Bayesian phylogenies and mean evolutionary rate were estimated for Dataset-2 by using the Bayesian Markov chain Monte Carlo (MCMC) sampler implemented in BEAST v1.8.3, using the HKY + G nucleotide substitution model and empirical base frequencies. Two demographic priors, constant size and Bayesian Skyline Plot (BSP), were compared by Bayes factors, enforcing in each case either a strict or relaxed (lognormal distributed rates) molecular clock. Proper mixing of the MCMC was achieved after running 1.5 billion generations and sampling every 150,000 generations. Proper mixing was assessed by calculating the effective sampling size (ESS) with Tracer v. 1.5 (http://tree.bio.ed.ac.uk/software/tracer/, accessed on 22 December 2021), with ESS > 200 for all parameters estimates (after 10% burn-in) considered acceptable. The maximum clade credibility (MCC) tree was chosen from the posterior distribution of sampled trees with TreeAnnotator v1.8.3 in the BEAST package, specifying a burn-in of 10% and median node heights [33]. Statistical support for specific clades was obtained by calculating the posterior probability of each monophyletic clade. Uncertainty in the estimates was indicated by 95% highest posterior density (95% HPD) intervals. The phylogenetic tree was edited graphically in FigTree v1.4.2 (available from http://tree.bio.ed.ac.uk/software/figtree/, accessed on 22 December 2021) for display purposes. Marginal likelihood estimates using path sampling and stepping-stone methods [37] of a different clock/demographic models were compared by Bayes Factors (BF), wherein a null hypothesis was compared to the alternative hypothesis as follows: 2lnBF < 2 indicates no evidence; 2–6-weak evidence; 6–10-strong evidence; and >10 indicates very strong evidence [38].

### 2.5. Metapopulation Analysis

HIV-1 gene flow (migration) events among distinct HIV-1 sub-populations within different geographic regions and cities in Bulgaria and neighboring countries were traced in Dataset-1 (1768 sequences) by most parsimonious reconstruction (MPR) of ancestral locations (internal nodes of the tree) followed by a count of migration events to/from different viral subpopulations, using the MacClade version 4 program (Sinauer Associates, Sunderland, MA, USA). Specific migrations among different countries (character states) were traced with the State changes and stasis tool (MacClade software), which counts the number of changes in a tree for each pair-wise character state. When multiple MPRs were present (as in our datasets), the algorithm calculated the average migration count over all possible MPRs for each pair. The resulting pair-wise migration matrix was then normalized, and a randomization test with 10,000 matrices obtained from 10,000 random trees (by random joining-splitting of the original tree) was performed to assess the statistical significance of the observed migration counts.

### 2.6. Ethics Statement

This study was approved by the Ethical Committee at the National Centre of Infectious and Parasitic Diseases, Sofia, Bulgaria (NCIPD IRB 00006384).

## 3. Results

### 3.1. Study Population Demographics

In this nationwide study, 37 individuals diagnosed with HIV/AIDS between 1986 and 2017 were analyzed. Men and women were equally represented with, respectively, 19 (51.4%) men and 18 (48.6%) women (Table 1). The majority of individuals *n* = 34 (91.9%) attributed their infection to heterosexual contact, while one individual (2.7%) reported he was MSM, one was a hemophiliac (2.7%) infected in the early years of the epidemic, and one was a newborn (2.7%) infected by vertical transmission (Table 1). The youngest individual in the study population was a newborn, the oldest was 62 years old, and the mean age of the population studied was 34.3 years. According to patient data, 28 (75.7%) were suspected of being infected in Bulgaria, whereas nine (24.3%) reported it likely that they were infected abroad while traveling or living outside the country in, for example, Germany (*n* = 2), South Africa (*n* = 1), or Congo (*n* = 1), while one patient was a refugee from Somalia. Four individuals did not indicate the alleged country where the infection occurred. Five of the individuals were diagnosed between 1986 and 1995, 11 between 1996 and 2005, and 21 between 2006 and 2017 (Table 1).

### 3.2. Introduction and Distribution of HIV-1 Subtype C in the Country

The first HIV/AIDS cases in Bulgaria were detected in 1986 in HET individuals and patients with hemophilia [21]. Later, one of them was tested for antiretroviral resistance and found to be infected with HIV-1 subtype C. During the first five years from 1986 to 1990, a total of 87 individuals were diagnosed with HIV-1 in the country (1986 *n* = 3, 1987 *n* = 34, 1988 *n* = 26, 1989 *n* = 17, and 1990 *n* = 7). In 15 of these patients, the virus was isolated and genotyped, and a wide variety of HIV-1 subtypes were found including five (33.3%) individuals infected with HIV-1 subtype C, followed by three (20.0%) with subtype A1, three (20.0%) with subtype B, and by one of each (6.7%) with subtype F1, H, CRF04_cpx, and one unclassified clade.

**Figure 1:**
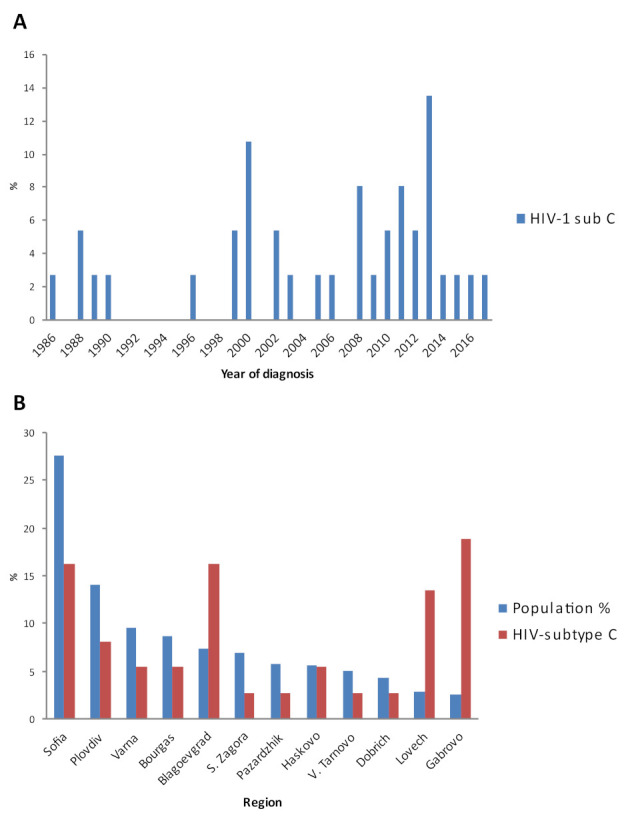
Distribution of HIV-1 subtype C in Bulgaria. (**A**) Number of individuals with HIV-1 subtype C by year of diagnosis. (**B**) Distribution of HIV-1 subtype C in different regions of the country. Population % represents the percentage of the population in the respective district of the country.

These results of the study indicate that subtype C was one of the first HIV-1 strains introduced in Bulgaria (Figure 1A). Additional epidemiological information on the residence of individuals infected with that particular strain reveals that HIV-1C has been spreading unevenly among distinct regions of the country (Figure 1B). Curiously, the largest cities were not the most affected, while there were specific areas of sparsely populated regions where HIV-1 subtype C was probably introduced by chance with a relatively larger number. One possible explanation could be the fact that HIV-1 subtype C is detected only in HET individuals with a single exception of one MSM, whereas the three major HIV-1 strains in the country, subtype B, CRF01_AE, and CRF02_AG, are found mostly in MSM and PWID, respectively.

### 3.3. Multiple Introductions of HIV-1 Subtype C in Bulgaria, Countries of Origin, and Transmission within the Country

The phylogenetic relationships of the Bulgarian and subtype reference sequences indicated that Bulgarian sequences were grouped into a separate phylogenetic cluster >99% ML bootstrap support, and the reference subtype C sequences branched of the Bulgarian cluster, indicating high phylogenetic similarity (Supplementary Figure S1).

**Figure 2:**
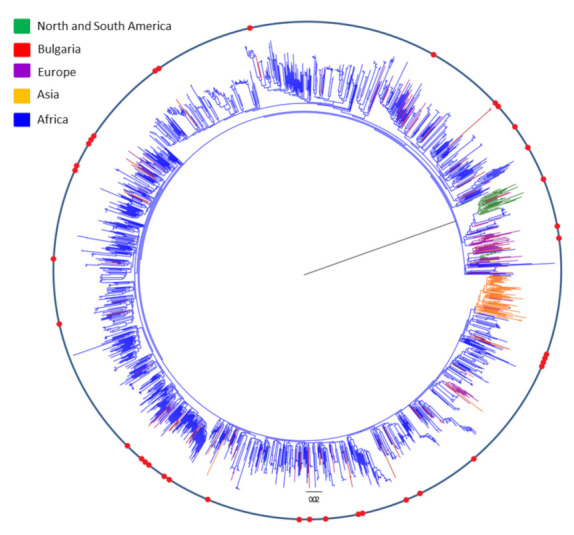
Global maximum likelihood phylogenetic tree of HIV-1 subtype C in Bulgaria. The tree reconstruction was inferred using Dataset-1 containing 37 Bulgarian sequences and 1731 specially selected HIV-1 subtype C sequences from the global search. A total of 1532 sequences originated from Africa, 98 from Europe, 67 from Asia, and 34 from North America and South America. The red dots on the circle indicate the position of the Bulgarian sequences on the phylogenetic tree. The colors of the branches of the phylogenetic tree correspond to the color chart on the relevant continent.

To further investigate the potential origin of HIV-1 subtype C in Bulgaria, we inferred alignment composed of 1768 globally selected HIV-1 *pol* sequences. This Dataset-1 combines 37 Bulgarian sequences together with 1731 specially selected HIV-1 subtype C sequences from the global search, 1532 of which originated from Africa, 98 from Europe, 67 from Asia, and 34 from North America and South America (Supplementary Table S1). An ML tree was then inferred to identify *pol* sequences from worldwide reference strains closely related to the Bulgarian isolates (Figure 2). Bulgarian sequences were dispersed across the entire phylogenetic tree, dismissing the scenario of a single founder event followed by regional dissemination but compatible with the hypothesis of multiple introductions from different countries over an extended period of time. The majority of the Bulgarian sequences (*n* = 21), isolated from 13 men and 8 women, were closely related to sequences from a different country from Africa, the Americas, Asia, and Europe, indicating multiple introductions followed by subsequent transmission to a limited number of partners. Only two highly supported (100% bootstrap) monophyletic clades with >2 Bulgarian sequences were identified in the ML tree. One cluster included strains isolated from two men and one woman from the Gabrovo and Lovech regions, while the other one was composed of sequences isolated from three women, all from the Blagoevgrad region. The phylogenetic tree also showed five highly supported (98–100% bootstrap) clades of related sequence pairs: two clades including pairs isolated from individuals in Sofia, two in Gabrovo, and one in Blagoevgrad.

### 3.4. Time-Scale of HIV-1 Subtype C Introductions in Bulgaria

**Figure 3:**
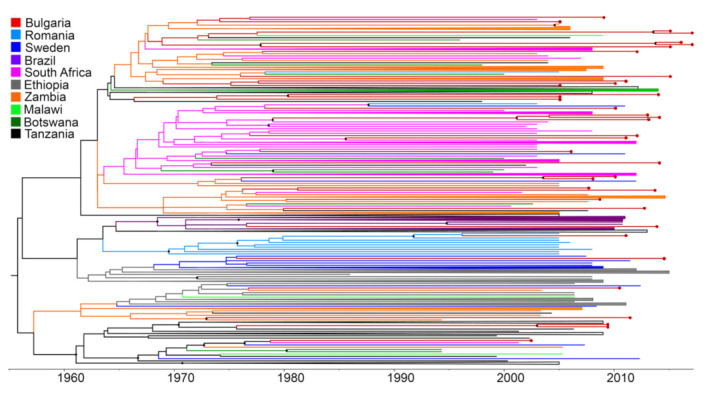
Bayesian molecular clock analysis of HIV-1 subtype C in Bulgaria. Bayesian maximum clade credibility tree and the time of the most recent common ancestor (tMRCA) estimation. The tree reconstruction was inferred using Dataset-2 consisting of 300 sequences, 37 of which were Bulgarian, and 263 were selected from the global search. Clades with posterior probabilities ≥90% are indicated by black dots. Red dots at the end of the red lines indicate Bulgarian sequences. Scale years are reported at the bottom of the figure. Some branches are collapsed for better visualization.

Bayesian time-scaled phylogenies were inferred from the HIV-1C reduced Dataset-2, which included all 37 new isolates, after checking the presence of sufficient temporal signal (root-to-tip distance vs. time regression and indicated positive slope in TempEst v1.5. program). Comparison of the marginal likelihoods of the MCMC runs under the different clock, and demographic models showed that the uncorrelated relaxed clock and BSP demographic priors were the ones best fitting the data (Supplementary Table S2). Thus, the maximum clade credibility tree was calculated from the posterior distribution of trees inferred with the selected model (Figure 3). The root of the tree traced back to the time of the most recent common ancestor (tMRCA) of the HIV-1C subtype, to the year 1954 (95% HPD: 1939–1966), which is in agreement with other estimates [15,39,40]. As expected, Bulgarian sequences were inter-dispersed in the tree due to numerous independent introductions from various geographic regions of the world.

**Figure 4:**
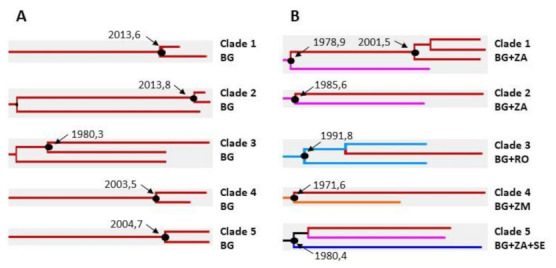
Statistically significant clusters with posterior probability >90% in the Bayesian maximum clade credibility tree. (**A**) Clusters involving sequences isolated in Bulgaria and (**B**) mixed clusters consisting of Bulgarian and non-Bulgarian sequences. Sequences from Bulgaria (BG) are represented in red color, South Africa (ZA) pink, Romania (RO) light blue, Zambia (ZM) orange, and Sweden (SE) dark blue.

The in-depth analysis revealed statistically significant (*p* > 0.9) clades containing only Bulgarian sequences as well as mixed clusters of Bulgarian and non-Bulgarian sequences (Figure 4A,B). Five clades contained only sequences isolated in Bulgaria (Figure 4A). Clade 1 included two sequences (both from women from different regions) with the tMRCA dating back to 2013.6 (95% HPD: 2010.1–2015.0); Clade 2 included three sequences (two from women and one from a man from two regions), tMRCA 2013.8 (95% HPD: 2010.4–2015.8); Clade 3 included two sequences (from women and man who have indicated they are partners), tMRCA 1980.3 (95% HPD: 1963.1–1991.5); Clade 4 included two sequences (from women and man who have indicated they are partners), tMRCA dating back to the year 2003.5 (95% HPD: 1996.6–2006.9); and Clade 5 included two sequences (from women and man who have indicated they are partners), tMRCA year 2004.7 (95% HPD: 1998.0–2008.4).

**Figure 5:**
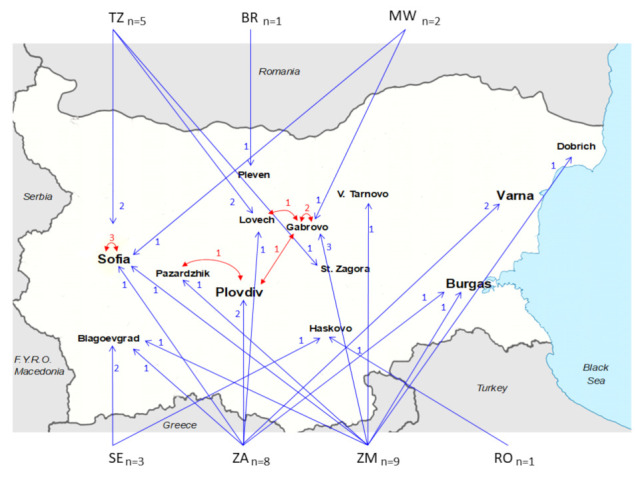
The introduction and dissemination of HIV-1 subtype C in Bulgaria. The blue arrows represent the introduction of strains from abroad into Bulgaria, and the red arrows represent the spreading of strains within the country. Country codes: ZM, Zambia; ZA, South Africa; TZ, Tanzania; SE, Sweden; MW, Malawi; BR, Brazil; RO, Romania. *n,* the number of the introduced strains in Bulgaria.

Statistically significant mixed clusters consisting of Bulgarian and non-Bulgarian sequences were also identified (Figure 4B). One of them included three Bulgarian sequences and one sequence from South Africa; one cluster included one sequence from Bulgaria and one from South Africa; one cluster included two sequences isolated from a neighboring country (Romania) and one Bulgarian sequence; one cluster included one sequence from Bulgaria and one from Zambia; and the last one included one sequence from Bulgaria, one from South Africa, and one from Sweden. Overall, the data showed that of all currently available HIV-1 subtype C strains isolated in Bulgaria, 29 (78.4%) were most likely introduced from abroad, while only eight (21.6%) were the result of subsequent local spread (Figure 5). The overall, phylogeographic dissemination pattern inferred from both ML and Bayesian analysis indicates at least seven countries as the likely source of multiple introductions of HIV-1 subtype C in Bulgaria. Most of the strains came from Zambia *n* = 9, followed by South Africa *n* = 8, Tanzania *n* = 5, Sweden *n* = 3, Malawi *n* = 2, Brazil *n* = 1, and the neighboring country of Romania *n* = 1 (Figure 5).

**Figure 6:**
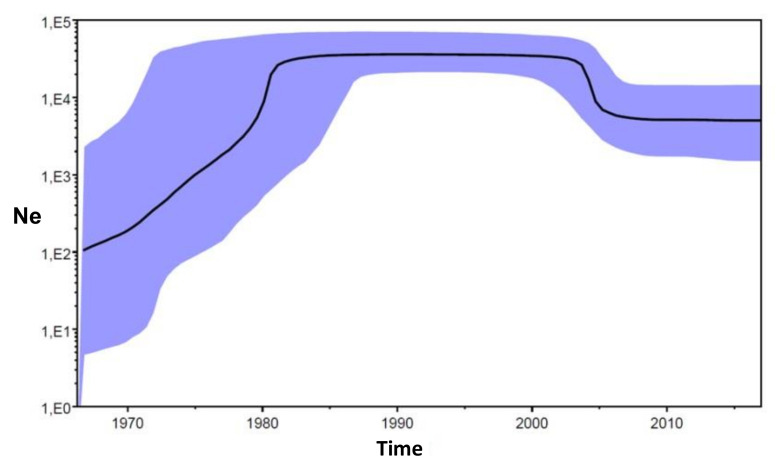
Bayesian skyline plot showing the inferred growth of the HIV-1 subtype C. Inferred effective population sizes (Ne) over time in years are on the y- and x-axes, respectively.

The coalescent-based population dynamics analysis of the isolates forming the HIV-1 subtype C clade was made using a non-parametric coalescent model-BSP to estimate past changes in effective population size. The BSP (Figure 6) demonstrates an upward and downward trend starting with an exponential phase from the late 1960s to 1980, followed by a constant phase up to about 2004 and a short decline followed by a constant phase.

## 4. Discussion

In this nationwide study, we analyzed the origin and introduction of HIV-1 subtype C, one of the early detected subtypes, in the HIV/AIDS epidemic in Bulgaria. The reconstruction of a Bayesian time-scaled phylogenies showed that several Bulgarian strains segregated together in small but highly significant clusters, while others were intermixed in larger clades containing strains isolated from both European and non-European countries. The analysis revealed the potential exporting countries from which the viral strains were introduced in Bulgaria. The present results extend those reported previously by our group that identified multiple subtypes in Bulgaria caused by viral inflow from European countries, USA, and Africa [22,24]. Our in-depth phylogenetic and phylogeographic analysis of all currently available HIV-1 subtype C sequences from Bulgarian patients is compatible with a scenario of multiple early introductions in the country during the 1970s, followed by limited local distribution in the subsequent years. Indeed, despite the limited availability of epidemiological data, about a quarter of individuals diagnosed during the period of this study (1986–2017) self-reported that they had likely acquired the infection abroad.

Available subtype C Bulgarian strains remained with limited distribution in the country and were almost exclusively detected in the heterosexual population and equally distributed among the sexes. In the phylogeny reconstruction, the clustering pattern of Bulgarian clearly indicated multiple introductions followed by transmission to a limited number of partners. This is probably related to the rapid and adequate measures taken by health authorities in the country in the late 1980s, with mass screening being conducted on large groups of the population, including Bulgarian citizens who had worked abroad. These measures allowed a significant number of infected individuals to be diagnosed for a short time in the early years of the epidemic (1987–1990). Urgent and large-scale measures have been taken by the Ministry of Health and the Government to limit the spread of HIV among the general population. The detection of a significant number of HIV-infected individuals in the first years and the subsequent decline in the number of new cases later showed that the virus was spread to a limited scale in Bulgaria without allowing underhanded development of the epidemic. However, the constant arrival of new strains from abroad raises concerns that these viruses could spill into vulnerable transmission groups, including MSM and PWIDs, causing outbreaks in these at-risk populations that were already reported with other viral strains, such as CRF01_AE and CRF02_AG, in our previous studies [21,24,25,41,42].

The analysis of the population dynamics represented on the Bayesian skyline plot showed a 2/3-log increase in the effective number of HIV-1 subtype C infections in between the second half of the 1960s (when the exponential growth began) and the early 1980s, when the plot reached a plateau until 2005, and then, the effective number of infections decreased, reaching a plateau that still persists today (Figure 6). This period can be coincident with a considerable policy of prevention, improving the number of safe medical procedures, such as blood transfusions and unsafe therapeutic injections, which are considered to be one of the causes of the spread of HIV together with unprotected sex [43]. The frequently asymptomatic infections induced by HIV probably helped to disguise the outbreak and its spread to other countries.

Interestingly, based on our previous and current analyses, the growth of the epidemic was not equally gradual for all HIV-1 subtypes, and Bulgaria experienced the explosive expansion of subtype B, CRF01_AE, and CRF_02_AG, mainly associated with MSM and PWIDs, which in turn are also most vulnerable to other bloodborne pathogens but not to subtype C, which was identified in HET individuals [44]. In fact, our epidemiological observations and phylodynamic analyses on the HIV/AIDS epidemic reviewed the lack of explosive spread of HIV-1 subtype C in Bulgaria, which could be explained by several possible reasons. First of all, the timely and large-scale efforts of health authorities to limit the spread of HIV/AIDS in the early years of the epidemic (when the first strains of this subtype were introduced) prevented its widespread dissemination. The second premise is that subtype C was introduced by heterosexual individuals who most often pass the virus only to their partners, and the infection ended in a dead-end without leading to ongoing transmission in the general or vulnerable populations. It is very likely for these reasons that the clusters formed by the Bulgarian isolates are composed of only a few sequences without an epidemic outbreak. In contrast, possibly by chance, other viral strains have come into populations at risk for blood- and sexually transmitted infections, such as PWIDs or MSM, which in turn quickly disseminated the virus [24,25]. However, our studies have already shown that there may be bridges between different groups, indicating that there is a risk of transmission of viral strains from one group to another, which gives rise to serious concerns.

## 5. Conclusions

In conclusion, the Bayesian framework, which allows a spatiotemporal reconstruction of phylogeny and an estimate of population dynamics using a coalescent-based approach, indicates that the first HIV-1 subtype C strains entered Bulgaria in the late 1970s and early 1980s as a result of multiple independent events. The intermixing clades indicate a different way of introductions over time, followed by the formation of clusters of the Bulgarian strains in separate monophyletic groups, suggesting the development of independent viral circulation of the HIV-1 subtype C epidemic in the country. It is important to note that only a small proportion of infections have been carried out within the country, most of which by means of a heterosexual road and probably as a consequence of the comprehensive actions for prevention and mass screening of the population in the early years of the HIV epidemic in Bulgaria.

## Figures and Tables

**Table 1:** Study populations characteristics.

Patients Characteristics	Patients with HIV-1 Subtype C	
	(*n*)	(%)
**Total**	37	100
Men	19	51.4
Women	18	48.6
**Age (years)**		
≤20	1	2.7
20–29	15	40.5
30–39	8	21.6
40–49	8	21.6
≥50	5	13.5
Mean age (years)	34.3	
**Likely country of infection**		
Bulgaria	28	75.7
Other country	9 (2 Germany, 1 Somalia, 1 South Africa, 1 Congo, 4 Uncnown)	24.3
**HET**	34	91.9
**MSM**	1	2.7
**Blood transfusion**	1	2.7
**Mother to child**	1	2.7
**Diagnosis period**		
1986–1995	5	13.5
1996–2005	11	29.7
2006–2017	21	56.8

## Data Availability

All sequences used in the study are publicly available from GenBank https://www.ncbi.nlm.nih.gov/genbank/. The dataset used in this study are available on request from the corresponding author.

## Supplementary Materials

The following supporting information can be downloaded at: https://www.mdpi.com/article/10.3390/v14020263/s1, Table S1. Specially selected HIV-1 subtype C sequences from the global search; Table S2. Bayes factor (BF) comparison of the nested molecular clock and Bayesian demographic models. The natural logarithm of the BF was used for comparison of the strict clock (SC) and uncorrelated relaxed lognormal clock (RC) models and the constant and non-parametric Bayesian skyline plot (BSP) demographic models within BEAST 1.8.3; Figure S1. Inferred phylogenetic relationships of Bulgarian HIV-1 and subtype reference sequences.
