# Supplementary material for: Analysis of the Origin and Dissemination of HIV-1 Subtype C in Bulgaria
Source: Viruses. 2022 Jan 27;14(2):263. doi: 10.3390/v14020263 (PMC8875591; doi:10.3390/v14020263)
Supplement: Supplementary file 1 [file viruses-14-00263-s001.zip › viruses-1546054-supplementary.pdf]

Table S1. *Specially selected HIV-1 subtype C sequences from the global search.*

Country code	Country	Sequence number	Continent
BW	BOTSWANA	76	Africa
ET	ETHIOPIA	24	Africa
KE	KENYA	5	Africa
MW	MALAWI	24	Africa
NG	NIGERIA	2	Africa
SN	SENEGAL	1	Africa
SO	SOMALIA	1	Africa
TZ	TANZANIA	77	Africa
ZA	SOUTH AFRICA	505	Africa
ZM	ZAMBIA	817	Africa
AR	Argentina	1	America
BR	BRAZIL	22	America
US	UNITED STATES	10	America
UY	URUGUAY	1	America
CN	CHINA	9	Asia
IL	ISRAEL	5	Asia
IN	INDIA	43	Asia
NP	NEPAL	8	Asia
PK	PAKISTAN	1	Asia
YE	YEMEN	1	Asia
CY	CYPRUS	12	Europe
DK	DENMARK	2	Europe
ES	SPAIN	9	Europe
GE	GEORGIA	1	Europe
RO	ROMANIA	27	Europe
SE	SWEDEN	47	Europe

Table S2. Bayes factor (BF) comparison of the nested molecular clock and Bayesian demographic models. The natural logarithm of the BF was used for comparison of the strict clock (SC) and uncorrelated relaxed lognormal clock (RC) models and the constant and non-parametric Bayesian skyline plot (BSP) demographic models within BEAST 1.8.3.

Molecular clock and demographic models	SS[4]	ln BF SS	PS[5]	ln BF PS
SC[1]<br>Constant	-29828,05672		-29821,75231	
**RC[2]**<br>**Constant**	**-29785,25666**	**42,8**	**-29780,02731**	**41,73**
RC[2]<br>Constant	-29785,25666		-29780,02731	
**RC[1]**<br>**BSP[3]**	**-29372,833**	**412,42**	**-29357,34271**	**422,68**
SC[1]<br>BSP[3]	-29411,54915		-29398,0643	
**RC[2]**<br>**BSP[3]**	**-29372,833**	**38,72**	**-29357,34271**	**40,72**

[1]SC = Strict Clock; [2]RC = Uncorrelated Relaxed Lognormal Clock; [3]BSP = Bayesian skyline plot; [4]SS = Stepping stone sampling; [5]BF = Bayes Factor; [6]PS = Path sampling

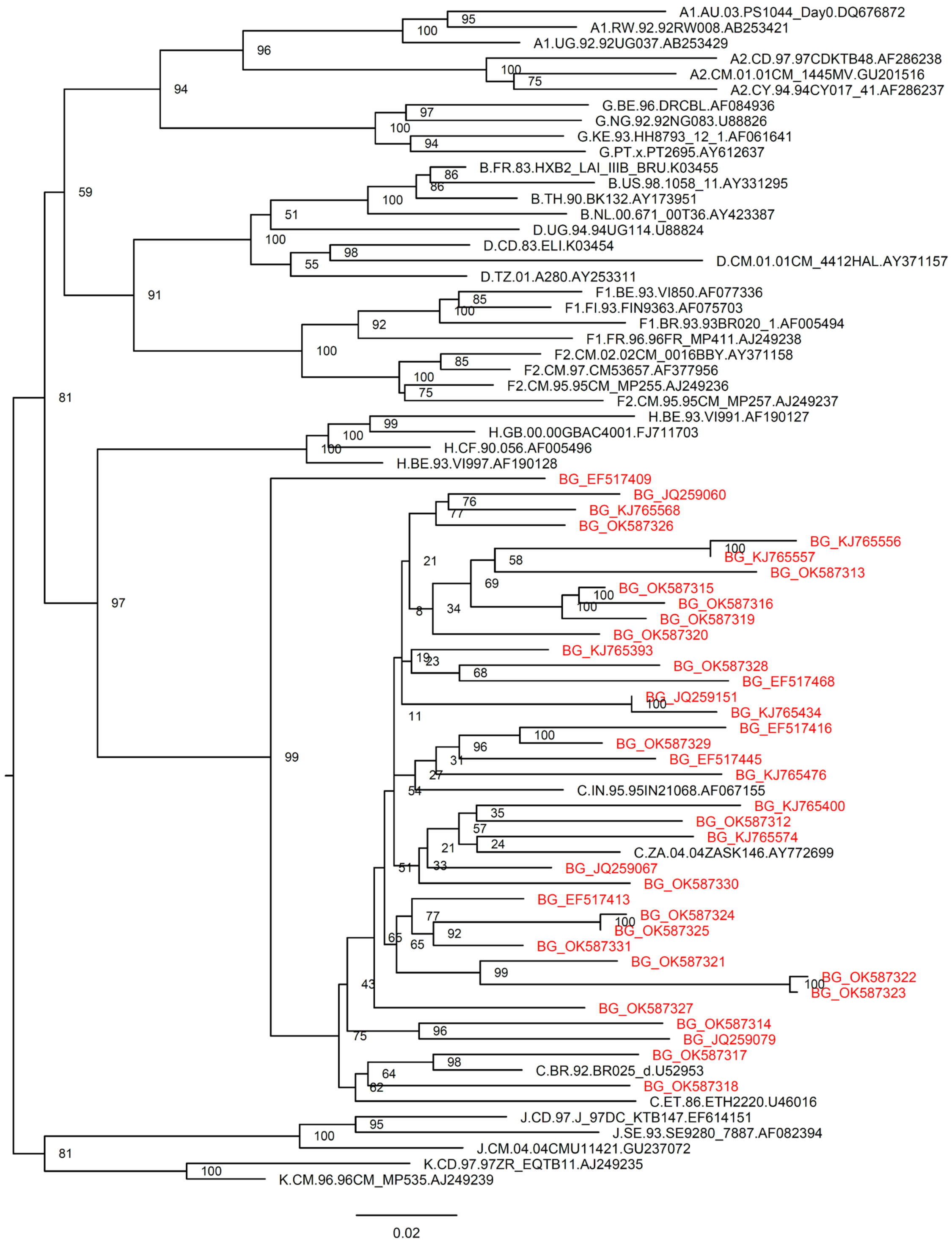

Figure S1. Inferred phylogenetic relationships of Bulgarian HIV-1 and subtype reference sequences. Tree reconstruction was inferred using ML analysis of polymerase sequences implemented in IQ-TREE. Support for each node was determined using ultrafast bootstrap with 1000 replicates. Scale bar at the bottom of the tree indicates the number of nucleotide substitutions per site. Antiretroviral resistance associated mutations were stripped from the alignments. The 723-bp alignment consisted of 37 HIV-1 subtype C strains from Bulgaria and 39 Group M reference sequences from the Los Alamos HIV database. The tree was rooted by using an HIV-1 subtype J strains as the outgroup. Bulgarian sequences are shown using red taxon names.
